# Perception, usage, and concerns of artificial intelligence applications among postgraduate dental students: cross-sectional study

**DOI:** 10.1186/s12909-025-07544-6

**Published:** 2025-06-23

**Authors:** Susan Sarhan, Amira Badran, Dalia Ghalwash, Hadeel Gamal Almalahy, Asmaa Abou-Bakr

**Affiliations:** 1 Oral Medicine and Periodontology, Ain Shams University in Egypt, Cairo, Egypt; 2 Pediatric Dentistry and Dental Public Health, Ain Shams University in Egypt, Cairo, Egypt; 3 Oral Medicine and Periodontology, Faculty of Dentistry, The British University, El Sherouk City, Egypt; 4 Oral Medicine and Periodontology, Faculty of Dentistry, Galala University, Suez, Egypt

**Keywords:** Artificial intelligence, Perception, Dental students, Usage, Concerns

## Abstract

**Background:**

Future dental applications of artificial intelligence (AI) are anticipated to be widely adopted across all dental specialities. However, there are some concerns among many users about the accuracy of the given information. Therefore, this study aimed to investigate postgraduate’ dental students’ perception, usage, and concerns towards AI systems’ applications.

**Materials and methods:**

An online self-administered survey, consisting of 19 closed-ended questions in the English language, and a 3-point Likert-type scale was used to obtain a simple and straightforward response from participants in a “forced-choice” response format that was distributed to postgraduate dental students in the faculty of dentistry of multiple Universities.

**Results:**

Younger participants and BDS holders are more likely to use AI-based software (*p* < 0.001), as well as showing more optimism about AI’s potential to advance dentistry, whereas PhD holders are more skeptical about its integral role in healthcare (*p* < 0.001). Speciality influenced AI adoption significantly, with Endodontics showing the highest percentage (52.4% for 1 + years of AI usage; *p* = 0.006). Concerns about AI reliability and originality in research vary significantly by level of education and Speciality (*p* < 0.05). Younger participants show greater belief in AI’s potential for major advancements in dentistry (*p* < 0.001).

**Conclusions:**

Postgraduate dental students generally perceive AI positively, recognizing its potential to enhance care. Usage remains moderate, with higher adoption in specialities like Endodontics and Periodontics. Concerns include AI’s accuracy, ethical implications, and integration challenges, highlighting the need for further education and research.

**Clinical trial number:**

Not applicable.

**Supplementary Information:**

The online version contains supplementary material available at 10.1186/s12909-025-07544-6.

## Introduction

The integration of artificial intelligence (AI) into dental practice and dental education has gained increased attention in the literature recently, highlighting the need for curricular reform, ethical safeguards, and practical validation frameworks [1, 2]. These advancements offer innovative tools that enhance diagnostic precision, treatment planning, and patient engagement [3]. In November 2022, a major leap in AI applications occurred with the public release of a large language model (LLM) capable of understanding and generating human-like responses across multiple languages. Built on neural network architectures, these systems analyze and respond to natural language with contextual awareness and adaptability, making them suitable for both clinical and academic settings [4].

Beyond general applications, AI technology is making transformative contributions to specific dental specialities, particularly in restorative, prosthodontic dentistry, as well as in surgical implant planning. For example, a recent in vitro study has demonstrated that AI-assisted intraoral scanners improve the accuracy of complete-arch digital impressions, particularly for implant-supported prostheses [5–9].

In implantology, AI-integrated guided surgery systems such as Smart Computer-Aided Implant Surgery (SCAIS) technology has shown promising results in optimizing implant positioning, minimizing surgical complexity and improving clinical outcomes [6]. Additionally, the integration of AI and augmented reality (AR) technologies could revolutionize 3D surgical planning and potentially replace traditional software [8].

Meanwhile, AI performance in detecting dental caries varies across different platforms; however, meta-analyses report higher sensitivity and comparable specificity to bitewing radiography, aiding clinicians in diagnosing caries from clinical images [7, 9]. These serve as examples of the very integration of AI technologies into clinical practice and the necessity of assessing the dental profession’s willingness to embrace such revolutionary applications.

While AI’s benefits are evident in automating routine tasks and enhancing productivity, ongoing debates highlight potential drawbacks. These include the propagation of biases embedded in training datasets, the phenomenon of “hallucinations” where plausible yet factually incorrect outputs are generated, and security vulnerabilities that could lead to misuse or misinformation [10, 11].

From a psychological perspective, resistance to technological change is common and understandable, particularly in healthcare where accuracy and ethics are paramount [12]. Innovations such as AI-driven image analysis and decision-support systems are reshaping clinical workflows, yet concerns persist regarding ethical implications, algorithmic transparency, and validation in real-world settings [13].

Contemporary AI systems leverage deep learning architectures to replicate cognitive processes such as pattern recognition, enhancing tasks like image analysis and decision-making [14]. In educational settings, AI holds promise for personalized learning experiences and virtual simulation. However, challenges such as limited faculty training, implementation costs, and unresolved ethical issues continue to hinder full-scale integration [15, 16]. With the growing presence of AI in medical and dental education, exploring students’ readiness and concerns is essential for informed curriculum design and technology adoption.

AI is rapidly reshaping the landscape of dental education system and clinical practice by providing advanced tools that enhance diagnostics, treatment planning, and decision-making processes. While several international studies have examined dental students’ perceptions of AI in countries such as India [17], Saudi Arabia [18] and South Korea [19], there remains a lack of large-scale, multi-institutional research within Egypt. One recent Egyptian study [20] assessed knowledge and attitude about AI among dental students, but it was limited by a smaller sample size and was confined to a single institution. In contrast, the current study offers a broader perspective by including a larger and more diverse sample of postgraduate dental students coming from multiple universities across Egypt.

Although AI applications have been widely incorporated into dental education and clinical practice in many parts of the world, their implementation in Egyptian dental institutions remains limited, with few formal programs or courses currently available. This disparity presents a timely opportunity for a more representative understanding of AI awareness, usage, and related concerns present within the Egyptian dental academic context, particularly at the postgraduate level, due to their advanced clinical experience and specialization, thus offering more relevant insights into AI applications in dentistry than undergraduates.

Given this context, the present study aims to investigate the perceptions, usage, and concerns related to AI applications among postgraduate dental students in Egypt. The objectives include assessing levels of awareness, evaluating engagement with AI tools, and identifying perceived barriers to adoption in both clinical and academic contexts. Understanding their attitudes and concerns will help inform strategies for integrating AI responsibly in dental education and practice.

## Methods

### Study design

This was an exploratory cross-sectional survey that utilized a self-administered online questionnaire distributed to postgraduate dental students across Egyptian universities to generate baseline data for future hypothesis-driven research. The survey structure was adapted from Roganović et al. [15], comprising 19 closed-ended questions in English on a 3-point Likert scale (Agree/Neutral/Disagree) to minimize neutral bias [15]. The tool was piloted with 30 students for clarity, yielding a Cronbach’s α of 0.78.

### Study timeline

This study was conducted through the academic year 2023–2024, during which the questionnaire was distributed to eligible postgraduate dental students across participating institutions.

### Ethical approval

The current study was conducted in accordance with the Helsinki Declaration. The study protocol was reviewed and approved by the Faculty of Dentistry, Ain Shams University research ethical committee with approval number (FDASU-REC ER112403).

### Sample size Estimation

For the sample size calculation, a power analysis was designed to have an adequate power by adopting an alpha (ɑ) level of (0.05), a beta (β) of (0.2) (i.e. power = 80%), and an effect size (d) of (1.63) calculated based on the results of a previous study by Kansal et al., [4]. The minimal required sample size (n) was found to be 620 surveys. The sample size was increased by 20% to compensate for possible excluded surveys to 744 surveys, which aligns with recommendations for attrition compensation [21]. Sample size calculation was performed using G*power version 3.1.9.7^39^ [22].

### Participants’ eligibility criteria

The survey was distributed to a total of 744 postgraduate dental students with the following criteria:

### Inclusion criteria

- Both genders.
- All participants should be post-graduate dental students.
- All participants must be Egyptians.

### Exclusion criteria

- Any undergraduate dental student.
- Postgraduate students who are unwilling to fill out the informed consent form and are reluctant to participate.
- Postgraduate students who did not answer all survey questions and had skipping histories.

The studied sample was restricted to Egyptian postgraduate dental students to ensure a representative reflection of the national dental education context. This approach minimizes variability arising from different curricula, clinical training, and access to AI technologies among students from other countries or educational systems.

### Sampling strategy

Consecutive sampling method was employed to recruit participants in the study. The questionnaire was distributed via email or any other means of digital communication platforms to all postgraduate dental students across multiple universities in Egypt who met the eligibility criteria including enrollment status and willingness to participate. Participation was voluntary and anonymous. The survey remained open until the predetermined sample size was reached based on the number of fully completed responses.

### Outcomes measure

The study aimed to assess:

1. Postgraduate dental students’ perceptions of AI and its role in dental practice.
2. The extent and nature of AI usage among participants.
3. Concerns and perceived barriers related to AI adoption in dental education and clinical practice.
4. The relationship between demographic variables and AI-related perceptions, usage, and concerns.

These outcomes were measured using a well-structured online questionnaire.

### Measurement instruments

A structured 19-item online questionnaire was developed, utilizing closed-ended questions and a 3-point Likert scale based on previous studies [4, 15, 20, 23, 24]. The purpose was to encourage participants to carefully consider their responses and to reduce the response bias that can occur when participants always select the neutral option [15, 20] as presented in Textbox 1 and Multimedia Appendix 1.

Based on the assumption that participants were able to compare the items and make relative judgments about them, even if they may not be able to provide precise or accurate ratings, we used the rank order scale [25].

The questionnaire was distributed to postgraduate dental students among the faculty of dentistry at many Universities in Egypt. Participation was voluntary and anonymous [26]. As the research project was clearly described in the note accompanying the questionnaire, the completion of the questionnaire by the respondents was regarded as their consent to participate in the study [27]. The questionnaire consisted of three parts that aimed to provide information on awareness of the usage of artificial intelligence in dental practice, perception, and concerns. The average completion time was 10–12 min.

To ensure data completeness, the survey was designed as a mandatory response questionnaire, requiring participants to answer all questions before submission, thereby ensuring that no responses were missing or incomplete.

### Questionnaire tool

Data were collected through a structured questionnaire consisting of multiple sections similar to previous studies [15, 23]:

1. **Demographic Data**: The first section gathered information about participants’ background, including gender, age group, years of experience, educational qualification (BDS, Master’s, or PhD), speciality, and institution of study. This section helped establish potential correlations between demographic factors and AI-related perceptions, usage, and concerns.
2. **Perception of AI**: This section focused on participants’ opinions regarding AI’s role in dentistry. Questions explored whether they believed AI would revolutionize dental practice, its potential benefits in improving efficiency, accuracy, and patient care, and whether AI could replace human dentists in the future. Additionally, it assessed their interest in learning AI concepts and their views on AI as a passing trend versus a transformative advancement in healthcare.
3. **Usage of AI**: This section examined participants’ familiarity and experience with AI-based tools in dentistry. Questions covered whether they had ever used AI applications, the duration of their usage, specific AI software they had encountered (e.g., ORCA Dental AI, Denti AI), and their participation in AI-related educational activities such as courses, webinars, or workshops. The section also identified gaps in AI education and professional exposure.
4. **Concerns About AI**: This section explored participants’ apprehensions regarding AI integration in dentistry. It assessed concerns about AI’s accuracy and reliability, its impact on clinical decision-making, ethical implications, over-reliance on technology, potential loss of originality in research, and the lack of clinical validation for AI applications. Additionally, it examined perceived barriers to AI adoption, such as low awareness among patients and practitioners.

Privacy and confidentiality were considered as none of the participants wrote his/her name or ID number, only codes to denote the number of participants were used. To avoid duplication, a note was added at the beginning of the questionnaire including “Please do not fill out this questionnaire again if you have previously done “.

### Data management

All data was entered electronically. Participants’ questionnaires were stored in numerical order in a secure place and with limited access files.

## Statistical analysis of the data

The data were entered into a computer for analysis using the IBM SPSS software package version 20.0 [21]. **(**Armonk, NY: IBM Corp, released 2011**)**. Categorical data were represented as numbers and percentages. **Chi-square test** was applied to assess associations between categorical variables, such as gender, age groups, educational qualifications and dental specialities, in relation to AI usage, perception, and concerns. Alternatively, **Fisher Exact test** or **Monte Carlo correction** was applied when more than 20% of the cells have an expected count of less than 5. Significance of the obtained results was judged at the 5% level. Multivariate logistic regression analysis was performed to identify independent predictors influencing participants’ perceptions and use of artificial intelligence (AI). Binary responses (Yes/No) to a variety of AI-related queries were among the dependent variables. The models included demographic and professional data such as gender, age group, education level, graduation year, and speciality as independent variables. To estimate the intensity and direction of associations, odds ratios (OR) with 95% confidence intervals (C.I.) were computed. When the p-value was less than 0.05, statistical significance was established. Version 20.0 of IBM SPSS Statistics for Windows (Armonk, NY: IBM Corp.) was used for all analyses.

## Results

### Demographic data of the study participants

Out of the 744 participants in the sample, 420 (56.5%) were female, and 324 (43.5%) were male. The largest age group was 20–25-year-olds (377, or 50.7%), followed by 30–35 years old (133, or 17.9%), 35–40 years old (123, or 16.5%), and 25–30 years old (111, or 14.9%). The majority of participants (62.8%) held a BDS degree, followed by PhDs (31.7%), and Master’s degrees 41 (5.5%). Over half graduated between 2020 and 2024 (51.3%). Periodontics was the most represented speciality (32.7%), while Oral & Maxillofacial Surgery was the least (1.6%). Ain Shams University contributed the highest proportion of participants (50.8%), with lower contributions from more recent universities like RUE (1.3%).

### Usage of AI

The majority (79.6%) had never used AI, while only 17.6% limited experience for less than a year. Only 21.4% reported using AI-based software such as ORCA Dental AI, Denti AI. Attendance at AI-related educational events was reported by 31.5% as attending webinars, seminars, or courses on AI, while 68.4% reported not participating in any educational activities. There is a notable awareness gap since a large portion (71.1%) have not used any resources to learn about AI.

**Perception of AI**:

Most participants (58.6%) believed AI will usher in a new era in dentistry, compared to 26.5% who disagree and 14.9% who are unsure. About half participants (50.3%) expressed interest in learning AI principles. While 42.9% of respondents think AI would be crucial to healthcare services, only 39.5% agreed that AI could enhance dentists’ effectiveness. Radiology (43.8%), Oral Medicine & Diagnosis (17.7%), and Endodontics (16.1%) were seen as the specialities most likely to benefit from AI. The majority (68.1%) doubt that AI might replace dentists, while only 4.4% thought it might.

### Concerns about AI

A majority of participants (83.2%), expressed concern about the accuracy of information produced by AI. Additional major concerns included the potential loss of originality in research (59.3%) and an overdependence on technology (78.1%). Furthermore, 77.4% of respondents highlighted the lack of clinical evidence supporting AI applications in dentistry. Limited awareness among patients (70.4%) and practitioners (62.8%) was also identified as a significant obstacle to effective AI implementation.

### Enhancing AI application in dentistry

Most participants (73.1%) emphasized the need for additional clinical trials to prove the viability of AI applications. Raising awareness among practitioners (65.9%) and patients (55%). Almost half (49.3%) suggested integrating AI into undergraduate or graduate dental education.

### Relations between AI usage duration and demographics

**Gender**: Males and females are equally represented across all usage categories, and no statistically significant association was found (*p* = 0.630). **Age**: The majority of participants who reported using AI for less than one year (84.7%) are in the youngest age group (20–25 years), whereas older participants are more likely to report never having used AI (*p* < 0.001). **Education**: Most short-term AI users (94.7%) hold a BDS degree, while non-users are more likely to have a PhD (38.3%, *p* < 0.001). **Speciality**: Endodontics has the highest percentage of AI utilization, with 52.4% of participants reported using AI for one or more years (*p* = 0.006), all are presented in Table 1.

**Table 1:** Relation between how many years have you been using AI for practice? With demographic data (*n* = 744)

Demographic data	How many years have you been using AI for practice?			χ^2^	*p*
	Less than 1 year (*n* = 131)	1 year or more (*n* = 21)	Never (*n* = 592)		
**Gender**					
Male	57 (43.5%)	7 (33.3%)	260 (43.9%)	0.924	0.630
Female	74 (56.5%)	14 (66.7%)	332 (56.1%)		
**Age (years)**					
20–25	111 (84.7%)	16 (76.2%)	250 (42.2%)	100.454^*^	^MC^p <0.001^*^
25–30	14 (10.7%)	1 (4.8%)	96 (16.2%)		
30–35	4 (3.1%)	3 (14.3%)	126 (21.3%)		
35–40	2 (1.5%)	1 (4.8%)	120 (20.3%)		
**Level of education**					
BDs	124 (94.7%)	18 (85.7%)	325 (54.9%)	77.629^*^	< 0.001^*^
Msc	1 (0.8%)	0 (0.0%)	40 (6.8%)		
PhD	6 (4.6%)	3 (14.3%)	227 (38.3%)		
**Graduation year**					
2005–2010	2 (1.5%)	1 (4.8%)	130 (22.0%)	100.470^*^	^MC^p <0.001^*^
2010–2015	4 (3.1%)	3 (14.3%)	125 (21.1%)		
2015–2020	14 (10.7%)	1 (4.8%)	82 (13.9%)		
2020–2024	111 (84.7%)	16 (76.2%)	255 (43.1%)		
**Specialty**					
Endodontics	36 (27.5%)	11 (52.4%)	102 (17.2%)	28.104^*^	^MC^*p*= 0.006^*^
Pedodontics	5 (3.8%)	2 (9.5%)	40 (6.8%)		
Oral- and maxillofacial surgery	1 (0.8%)	0 (0.0%)	11 (1.9%)		
Prosthodontics	1 (0.8%)	0 (0.0%)	35 (5.9%)		
Conservative Dentistry	3 (2.3%)	0 (0.0%)	31 (5.2%)		
Periodontics	44 (33.6%)	5 (23.8%)	194 (32.8%)		
Oral medicine and diagnosis	22 (16.8%)	1 (4.8%)	103 (17.4%)		
Oral Radiology	19 (14.5%)	2 (9.5%)	76 (12.8%)		

χ^2^: Chi square test MC: Monte Carlo

p: p value for Relation between How many years have you been using AI for practice? with demographic data

*: Statistically significant at *p* ≤ 0.05

### Relation between AI software usage and demographics

**Gender**: There was no statistically significant difference between male and female participants in AI software usage (*p* = 0.686). **Age**: AI software adoption is significantly lower among older age groups (*p* < 0.001), while younger participants (20–25 years) are more likely to use such software (81.1%). **Education**: A majority of AI software users (91.8%) hold a BDS degree, whereas non-users are more likely to have a PhD (38.5%, *p* < 0.001). **Speciality**: Periodontics (34.0%) and Endodontics (29.6%) show the highest rates of AI software usage, with significant differences observed across specialities (*p* = 0.004), all are presented in Table 2.

**Table 2:** Relation between do you use any of the current applications of AI-based dental software? With demographic data (*n* = 744)

Demographic data	Do you use any of the current applications of AI-based dental software?		χ^2^	*p*
	Yes (*n* = 159)	No (*n* = 585)		
**Gender**				
Male	67 (42.1%)	257 (43.9%)	0.164	0.686
Female	92 (57.9%)	328 (56.1%)		
**Age (years)**				
20–25	129 (81.1%)	248 (42.4%)	80.034^*^	< 0.001^*^
25–30	17 (10.7%)	94 (16.1%)		
30–35	8 (5.0%)	125 (21.4%)		
35–40	5 (3.1%)	118 (20.2%)		
**Level of education**				
BDs	146 (91.8%)	321 (54.9%)	73.049^*^	< 0.001^*^
Msc	2 (1.3%)	39 (6.7%)		
PhD	11 (6.9%)	225 (38.5%)		
**Graduation year**				
2005–2010	5 (3.1%)	128 (21.9%)	78.763^*^	< 0.001^*^
2010–2015	8 (5.0%)	124 (21.2%)		
2015–2020	17 (10.7%)	80 (13.7%)		
2020–2024	129 (81.1%)	253 (43.2%)		
**Specialty**				
Endodontics	47 (29.6%)	102 (17.4%)	20.621^*^	0.004^*^
Pedodontics	7 (4.4%)	40 (6.8%)		
Oral- and maxillofacial surgery	1 (0.6%)	11 (1.9%)		
Prosthodontics	2 (1.3%)	34 (5.8%)		
Conservative Dentistry	3 (1.9%)	31 (5.3%)		
Periodontics	54 (34.0%)	189 (32.3%)		
Oral medicine and diagnosis	24 (15.1%)	102 (17.4%)		
Oral Radiology	21 (13.2%)	76 (13.0%)		

χ^2^: Chi square test

p: p value for Relation between Do you use any of the current applications of AI-based dental software? with demographic data

*: Statistically significant at *p* ≤ 0.05

### Relation between AI education and demographics

**Gender**: There was no statistically significant difference in attendance at AI-related educational sessions based on gender (*p* = 0.739). **Age**: Attendance was significantly higher among participants aged 20–25 years (72.2%, *p* < 0.001). **Education**: Most attended participants (82.5%) were BDS holders, whereas PhD holders were more likely to have never attended any related AI-related sessions (40.5%, *p* < 0.001). **Speciality**: Participants in Endodontics showed the highest attendance rate (26.9%), with significant differences observed across specialities (*p* = 0.005), as presented in Table 3.

**Table 3:** Relation between have you ever attended any webinar/lecture/course on artificial intelligence in healthcare? With demographic data **(*n* = 744)**

Demographic data	Have you ever attended any webinar/lecture/course on Artificial Intelligence in healthcare?			χ^2^	*p*
	Yes (*n* = 234)	No (*n* = 509)	Maybe (*n* = 1)		
**Gender**					
Male	98 (41.9%)	226 (44.4%)	0 (0.0%)	1.121	^MC^p= 0.739
Female	136 (58.1%)	283 (55.6%)	1 (100.0%)		
**Age (years)**					
20–25	169 (72.2%)	207 (40.7%)	1 (100.0%)	80.715^*^	^MC^*p* < 0.001^*^
25–30	29 (12.4%)	82 (16.1%)	0 (0.0%)		
30–35	26 (11.1%)	107 (21.0%)	0 (0.0%)		
35–40	10 (4.3%)	113 (22.2%)	0 (0.0%)		
**Level of education**					
BDs	193 (82.5%)	273 (53.6%)	1 (100.0%)	67.255^*^	^MC^*p* < 0.001^*^
Msc	11 (4.7%)	30 (5.9%)	0 (0.0%)		
PhD	30 (12.8%)	206 (40.5%)	0 (0.0%)		
**Graduation year**					
2005–2010	14 (6.0%)	119 (23.4%)	0 (0.0%)	70.959^*^	^MC^*p* < 0.001^*^
2010–2015	29 (12.4%)	103 (20.2%)	0 (0.0%)		
2015–2020	22 (9.4%)	75 (14.7%)	0 (0.0%)		
2020–2024	169 (72.2%)	212 (41.7%)	1 (100.0%)		
**Specialty**					
Endodontics	63 (26.9%)	85 (16.7%)	1 (100.0%)	30.169^*^	^MC^p= 0.005^*^
Pedodontics	11 (4.7%)	36 (7.1%)	0 (0.0%)		
Oral- and maxillofacial surgery	1 (0.4%)	11 (2.2%)	0 (0.0%)		
Prosthodontics	5 (2.1%)	31 (6.1%)	0 (0.0%)		
Conservative Dentistry	7 (3.0%)	27 (5.3%)	0 (0.0%)		
Periodontics	79 (33.8%)	164 (32.2%)	0 (0.0%)		
Oral medicine and diagnosis	36 (15.4%)	90 (17.7%)	0 (0.0%)		
Oral Radiology	32 (13.7%)	65 (12.8%)	0 (0.0%)		

χ^2^: Chi square test MC: Monte Carlo

p: p value for Relation between Have you ever attended any webinar/lecture/course on Artificial Intelligence in healthcare? with demographic data

*: Statistically significant at *p* ≤ 0.05

### Perception of AI as a new era in dentistry

**Age**: Participants aged 20–25 are the most optimistic, with 65.8% agreeing that AI represents a new era in dentistry, while those aged 35–40 are the most skeptical (*p* < 0.001). **Education**: PhD holders express the greatest uncertainty, with 54.8% disagreeing (*p* < 0.001), whereas BDS holders are the most optimistic (75.2%). **Speciality**: Participants in Periodontics and Endodontics are more likely to view AI as transformative development (*p* = 0.001), all are presented in Table 4.

**Table 4:** Relation between do you think AI is a new era in dentistry? With demographic data (*n* = 744)

Demographic data	Do you think AI is a new era in dentistry?			χ^2^	*p*
	Yes (*n* = 436)	No (*n* = 197)	Maybe (*n* = 111)		
**Gender**					
Male	200 (45.9%)	87 (44.2%)	37 (33.3%)	5.699	0.058
Female	236 (54.1%)	110 (55.8%)	74 (66.7%)		
**Age (years)**					
20–25	287 (65.8%)	54 (27.4%)	36 (32.4%)	114.906^*^	< 0.001^*^
25–30	62 (14.2%)	34 (17.3%)	15 (13.5%)		
30–35	50 (11.5%)	55 (27.9%)	28 (25.2%)		
35–40	37 (8.5%)	54 (27.4%)	32 (28.8%)		
**Level of education**					
BDs	328 (75.2%)	85 (43.1%)	54 (48.6%)	123.227^*^	< 0.001^*^
Msc	37 (8.5%)	4 (2.0%)	0 (0.0%)		
PhD	71 (16.3%)	108 (54.8%)	57 (51.4%)		
**Graduation year**					
2005–2010	38 (8.7%)	53 (26.9%)	42 (37.8%)	119.643^*^	< 0.001^*^
2010–2015	60 (13.8%)	52 (26.4%)	20 (18.0%)		
2015–2020	49 (11.2%)	37 (18.8%)	11 (9.9%)		
2020–2024	289 (66.3%)	55 (27.9%)	38 (34.2%)		
**Specialty**					
Endodontics	97 (22.2%)	26 (13.2%)	26 (23.4%)	36.919^*^	0.001^*^
Pedodontics	19 (4.4%)	18 (9.1%)	10 (9.0%)		
Oral- and maxillofacial surgery	6 (1.4%)	3 (1.5%)	3 (2.7%)		
Prosthodontics	17 (3.9%)	12 (6.1%)	7 (6.3%)		
Conservative Dentistry	11 (2.5%)	13 (6.6%)	10 (9.0%)		
Periodontics	157 (36.0%)	61 (31.0%)	25 (22.5%)		
Oral medicine and diagnosis	66 (15.1%)	38 (19.3%)	22 (19.8%)		
Oral Radiology	63 (14.4%)	26 (13.2%)	8 (7.2%)		

χ^2^: Chi square test

p: p value for Relation between Do you think AI is a new era in dentistry? with demographic data

*: Statistically significant at *p* ≤ 0.05

### Perception of AI as a trend

**Gender**: Women are more likely to answer “maybe” (65.9%, *p* < 0.001), and men are more likely to think that AI is just a trend (53.0%). **Age**: Compared to younger respondents, older participants (35–40 years old) are more likely to think that AI is just a trend (31.9%) (*p* < 0.001). **Education**: A majority of PhD holders (87.7%) disagree that AI is only a trend (*p* < 0.001). **Speciality**: Participants in Periodontics and Oral Medicine & Diagnosis are less likely to disagree with the statement that AI is just a trend (*p* < 0.001), all are presented in Table 5.

**Table 5:** Relation between do you think AI is just a new trend? With demographic data (*n* = 744)

Demographic data	Do you think AI is just a new trend?			χ^2^	*p*
	Yes (*n* = 251)	No (*n* = 203)	Maybe (*n* = 290)		
**Gender**					
Male	133 (53.0%)	92 (45.3%)	99 (34.1%)	19.804^*^	< 0.001^*^
Female	118 (47.0%)	111 (54.7%)	191 (65.9%)		
**Age (years)**					
20–25	71 (28.3%)	150 (73.9%)	156 (53.8%)	132.353^*^	< 0.001^*^
25–30	43 (17.1%)	34 (16.7%)	34 (11.7%)		
30–35	57 (22.7%)	12 (5.9%)	64 (22.1%)		
35–40	80 (31.9%)	7 (3.4%)	36 (12.4%)		
**Level of education**					
BDs	110 (43.8%)	178 (87.7%)	179 (61.7%)	114.420^*^	< 0.001^*^
Msc	11 (4.4%)	15 (7.4%)	15 (5.2%)		
PhD	130 (51.8%)	10 (4.9%)	96 (33.1%)		
**Graduation year**					
2005–2010	80 (31.9%)	7 (3.4%)	46 (15.9%)	138.413^*^	< 0.001^*^
2010–2015	54 (21.5%)	12 (5.9%)	66 (22.8%)		
2015–2020	46 (18.3%)	32 (15.8%)	19 (6.6%)		
2020–2024	71 (28.3%)	152 (74.9%)	159 (54.8%)		
**Specialty**					
Endodontics	36 (14.3%)	57 (28.1%)	56 (19.3%)	41.344^*^	< 0.001^*^
Pedodontics	18 (7.2%)	10 (4.9%)	19 (6.6%)		
Oral- and maxillofacial surgery	9 (3.6%)	3 (1.5%)	0 (0.0%)		
Prosthodontics	14 (5.6%)	5 (2.5%)	17 (5.9%)		
Conservative Dentistry	15 (6.0%)	8 (3.9%)	11 (3.8%)		
Periodontics	83 (33.1%)	73 (36.0%)	87 (30.0%)		
Oral medicine and diagnosis	44 (17.5%)	18 (8.9%)	64 (22.1%)		
Oral Radiology	32 (12.7%)	29 (14.3%)	36 (12.4%)		

χ^2^: Chi square test

p: p value for Relation between Do you think AI is just a new trend? with demographic data

*: Statistically significant at *p* ≤ 0.05

### Interest in learning AI principles

**Age**: Participants aged 20–25 years exhibit the highest level of interest (64.4%), while those aged 35–40 years show the lowest level of interest (29.9%, *p* < 0.001). **Education**: PhD holders are less interested (53.5% disagree, *p* < 0.001), whereas BDS holders are the most interested (75.4%). **Speciality**: Participants in Endodontics showed the highest level of interest in understanding AI concepts (22.7%, *p* = 0.008), as presented in Table 6.

**Table 6:** Relation between do you feel interested in learning the principles of artificial intelligence and its applications in healthcare? With demographic data (*n* = 744)

Demographic data	Do you feel interested in learning the principles of Artificial Intelligence and its applications in healthcare?			χ^2^	*p*
	Yes (*n* = 374)	No (*n* = 271)	Maybe (*n* = 99)		
**Gender**					
Male	158 (42.2%)	117 (43.2%)	49 (49.5%)	1.698	0.428
Female	216 (57.8%)	154 (56.8%)	50 (50.5%)		
**Age (years)**					
20–25	241 (64.4%)	83 (30.6%)	53 (53.5%)	100.026^*^	< 0.001^*^
25–30	58 (15.5%)	45 (16.6%)	8 (8.1%)		
30–35	46 (12.3%)	62 (22.9%)	25 (25.3%)		
35–40	29 (7.8%)	81 (29.9%)	13 (13.1%)		
**Level of education**					
BDs	282 (75.4%)	126 (46.5%)	59 (59.6%)	132.877^*^	< 0.001^*^
Msc	39 (10.4%)	0 (0.0%)	2 (2.0%)		
PhD	53 (14.2%)	145 (53.5%)	38 (38.4%)		
**Graduation year**					
2005–2010	33 (8.8%)	87 (32.1%)	13 (13.1%)	105.126^*^	< 0.001^*^
2010–2015	47 (12.6%)	58 (21.4%)	27 (27.3%)		
2015–2020	48 (12.8%)	43 (15.9%)	6 (6.1%)		
2020–2024	246 (65.8%)	83 (30.6%)	53 (53.5%)		
**Specialty**					
Endodontics	85 (22.7%)	40 (14.8%)	24 (24.2%)	29.915^*^	0.008^*^
Pedodontics	17 (4.5%)	20 (7.4%)	10 (10.1%)		
Oral- and maxillofacial surgery	2 (0.5%)	9 (3.3%)	1 (1.0%)		
Prosthodontics	14 (3.7%)	15 (5.5%)	7 (7.1%)		
Conservative Dentistry	14 (3.7%)	19 (7.0%)	1 (1.0%)		
Periodontics	129 (34.5%)	87 (32.1%)	27 (27.3%)		
Oral medicine and diagnosis	60 (16.0%)	48 (17.7%)	18 (18.2%)		
Oral Radiology	53 (14.2%)	33 (12.2%)	11 (11.1%)		

χ^2^: Chi square test

p: p value for Relation between Do you feel interested in learning the principles of Artificial Intelligence and its applications in healthcare? with demographic data

*: Statistically significant at *p* ≤ 0.05

### AI’s role in future healthcare delivery

**Age**: The youngest group (20–25 years: 64.6%, *p* < 0.001) is the most optimistic. **Education**: PhD holders exhibit a higher level of doubt (55.4% disagree, *p* < 0.001), while BDS holders once again showed the highest level of optimism (76.2%). **Speciality**: Participants from the field of Endodontics and Periodontics expressed the strongest beliefs about the future of AI (*p* < 0.001), as presented in Table 7.

**Table 7:** Relation between do you think that “artificial intelligence will play an integral role in delivering healthcare services in the future”? With demographic data (*n* = 744)

Demographic data	Do you think that “Artificial Intelligence will play an integral role in delivering healthcare services in the future”?			χ^2^	*p*
	Yes (*n* = 319)	No (*n* = 269)	Maybe (*n* = 156)		
**Gender**					
Male	132 (41.4%)	114 (42.4%)	78 (50.0%)	3.401	0.183
Female	187 (58.6%)	155 (57.6%)	78 (50.0%)		
**Age (years)**					
20–25	206 (64.6%)	79 (29.4%)	92 (59.0%)	116.984^*^	< 0.001^*^
25–30	56 (17.6%)	43 (16.0%)	12 (7.7%)		
30–35	39 (12.2%)	62 (23.0%)	32 (20.5%)		
35–40	18 (5.6%)	85 (31.6%)	20 (12.8%)		
**Level of education**					
BDs	243 (76.2%)	120 (44.6%)	104 (66.7%)	169.203^*^	< 0.001^*^
Msc	41 (12.9%)	0 (0.0%)	0 (0.0%)		
PhD	35 (11.0%)	149 (55.4%)	52 (33.3%)		
**Graduation year**					
2005–2010	24 (7.5%)	92 (34.2%)	17 (10.9%)	125.357^*^	< 0.001^*^
2010–2015	41 (12.9%)	57 (21.2%)	34 (21.8%)		
2015–2020	51 (16.0%)	41 (15.2%)	5 (3.2%)		
2020–2024	203 (63.6%)	79 (29.4%)	100 (64.1%)		
**Specialty**					
Endodontics	66 (20.7%)	38 (14.1%)	45 (28.8%)	54.408^*^	< 0.001^*^
Pedodontics	16 (5.0%)	19 (7.1%)	12 (7.7%)		
Oral- and maxillofacial surgery	0 (0.0%)	10 (3.7%)	2 (1.3%)		
Prosthodontics	6 (1.9%)	16 (5.9%)	14 (9.0%)		
Conservative Dentistry	16 (5.0%)	18 (6.7%)	0 (0.0%)		
Periodontics	110 (34.5%)	89 (33.1%)	44 (28.2%)		
Oral medicine and diagnosis	57 (17.9%)	41 (15.2%)	28 (17.9%)		
Oral Radiology	48 (15.0%)	38 (14.1%)	11 (7.1%)		

χ^2^: Chi square test

p: p value for Relation between Do you think that “Artificial Intelligence will play an integral role in delivering healthcare services in the future”? with demographic data

*: Statistically significant at *p* ≤ 0.05

### AI’s impact on dentists’ efficacy

**Age**: While older participants (35–40 years) are more likely to disagree (35.3%, *p* < 0.001), younger respondents (20–25 years) are more likely to agree (64.6%) that AI enhances efficacy. **Education**: PhD holders are less optimistic (58.6% disagree, *p* < 0.001), compared to BDS holders (76.9%). **Speciality**: Participants in Endodontics and Periodontics are more likely to agree with AI’s ability to improve efficacy (*p* < 0.001), all are presented in Table 8.

**Table 8:** Relation between do you think AI could improve the efficacy of dentists in dental practice? With demographic data (*n* = 744)

Demographic data	Do you think AI could improve the efficacy of dentists in dental practice?			χ^2^	*p*
	Yes (*n* = 294)	No (*n* = 232)	Maybe (*n* = 218)		
**Gender**					
Male	129 (43.9%)	101 (43.5%)	94 (43.1%)	0.029	0.985
Female	165 (56.1%)	131 (56.5%)	124 (56.9%)		
**Age (years)**					
20–25	190 (64.6%)	55 (23.7%)	132 (60.6%)	136.261^*^	< 0.001^*^
25–30	49 (16.7%)	39 (16.8%)	23 (10.6%)		
30–35	39 (13.3%)	56 (24.1%)	38 (17.4%)		
35–40	16 (5.4%)	82 (35.3%)	25 (11.5%)		
**Level of education**					
BDs	226 (76.9%)	93 (40.1%)	148 (67.9%)	133.050^*^	< 0.001^*^
Msc	29 (9.9%)	3 (1.3%)	9 (4.1%)		
PhD	39 (13.3%)	136 (58.6%)	61 (28.0%)		
**Graduation year**					
2005–2010	21 (7.1%)	87 (37.5%)	25 (11.5%)	131.983^*^	< 0.001^*^
2010–2015	44 (15.0%)	53 (22.8%)	35 (16.1%)		
2015–2020	37 (12.6%)	37 (15.9%)	23 (10.6%)		
2020–2024	192 (65.3%)	55 (23.7%)	135 (61.9%)		
**Specialty**					
Endodontics	52 (17.7%)	32 (13.8%)	65 (29.8%)	43.515^*^	< 0.001^*^
Pedodontics	15 (5.1%)	24 (10.3%)	8 (3.7%)		
Oral- and maxillofacial surgery	2 (0.7%)	7 (3.0%)	3 (1.4%)		
Prosthodontics	14 (4.8%)	13 (5.6%)	9 (4.1%)		
Conservative Dentistry	12 (4.1%)	18 (7.8%)	4 (1.8%)		
Periodontics	110 (37.4%)	68 (29.3%)	65 (29.8%)		
Oral medicine and diagnosis	46 (15.6%)	42 (18.1%)	38 (17.4%)		
Oral Radiology	43 (14.6%)	28 (12.1%)	26 (11.9%)		

χ^2^: Chi square test

p: p value for Relation between Do you think AI could improve the efficacy of dentists in dental practice? with demographic data

*: Statistically significant at *p* ≤ 0.05

### AI advancements in specialities

**Age**: Those aged 20–25 show the highest levels of optimism (60.9%), while those aged 35–40 exhibit the highest levels of doubt (*p* < 0.001). **Education**: PhD holders appear more reserved (48.5% disagree, *p* < 0.001), whereas BDS holders are more optimistic (73.9%). **Speciality**: Participants in Periodontics show the strongest support for AI’s potential for advancement (37.9%, *p* = 0.001), as presented in Table 9.

**Table 9:** Relation between do you think that AI would facilitate major advancements related to your specialty? With demographic data **(*n* = 744)**

Demographic data	Do you think that AI would facilitate major advancements related to your specialty?			χ^2^	*p*
	Yes (*n* = 253)	No (*n* = 272)	Maybe (*n* = 219)		
**Gender**					
Male	117 (46.2%)	125 (46.0%)	82 (37.4%)	4.710	0.095
Female	136 (53.8%)	147 (54.0%)	137 (62.6%)		
**Age (years)**					
20–25	154 (60.9%)	101 (37.1%)	122 (55.7%)	72.643^*^	< 0.001^*^
25–30	46 (18.2%)	39 (14.3%)	26 (11.9%)		
30–35	46 (18.2%)	57 (21.0%)	30 (13.7%)		
35–40	7 (2.8%)	75 (27.6%)	41 (18.7%)		
**Level of education**					
BDs	187 (73.9%)	140 (51.5%)	140 (63.9%)	115.065^*^	< 0.001^*^
Msc	35 (13.8%)	0 (0.0%)	6 (2.7%)		
PhD	31 (12.3%)	132 (48.5%)	73 (33.3%)		
**Graduation year**					
2005–2010	15 (5.9%)	77 (28.3%)	41 (18.7%)	55.252^*^	< 0.001^*^
2010–2015	48 (19.0%)	54 (19.9%)	30 (13.7%)		
2015–2020	34 (13.4%)	37 (13.6%)	26 (11.9%)		
2020–2024	156 (61.7%)	104 (38.2%)	122 (55.7%)		
**Specialty**					
Endodontics	48 (19.0%)	40 (14.7%)	61 (27.9%)	35.123^*^	0.001^*^
Pedodontics	12 (4.7%)	26 (9.6%)	9 (4.1%)		
Oral- and maxillofacial surgery	0 (0.0%)	9 (3.3%)	3 (1.4%)		
Prosthodontics	10 (4.0%)	17 (6.3%)	9 (4.1%)		
Conservative Dentistry	10 (4.0%)	16 (5.9%)	8 (3.7%)		
Periodontics	96 (37.9%)	81 (29.8%)	66 (30.1%)		
Oral medicine and diagnosis	40 (15.8%)	47 (17.3%)	39 (17.8%)		
Oral Radiology	37 (14.6%)	36 (13.2%)	24 (11.0%)		

χ^2^: Chi square test

p: p value for Relation between Do you think that AI would facilitate major advancements related to your specialty? with demographic data

*: Statistically significant at *p* ≤ 0.05

### AI replacing dentists in the future

**Gender**: Men are more likely than women to believe that AI could eventually replace dentists (60.6% agree, *p* = 0.030). **Age**: Compared to younger participants, those between the ages of 25 and 30 are the most anxious (33.3%) (*p* = 0.001). **Education**: PhD holders are more likely to disagree (61.7%, *p* < 0.001), while BDS holders show moderate agreement. **Speciality**: Individuals in Periodontics show greater agreement with this concern (51.5%, *p* < 0.001), as presented in Table 10.

**Table 10:** Relation between do you think AI could replace dentists in the future? With demographic data (*n* = 744)

Demographic data	Do you think AI could replace dentists in the future?			χ^2^	*p*
	Yes (*n* = 33)	No (*n* = 507)	Maybe (*n* = 204)		
**Gender**					
Male	20 (60.6%)	227 (44.8%)	77 (37.7%)	7.010^*^	0.030^*^
Female	13 (39.4%)	280 (55.2%)	127 (62.3%)		
**Age (years)**					
20–25	14 (42.4%)	243 (47.9%)	120 (58.8%)	23.332^*^	0.001^*^
25–30	11 (33.3%)	81 (16.0%)	19 (9.3%)		
30–35	8 (24.2%)	93 (18.3%)	32 (15.7%)		
35–40	0 (0.0%)	90 (17.8%)	33 (16.2%)		
**Level of education**					
BDs	20 (60.6%)	313 (61.7%)	134 (65.7%)	35.650^*^	< 0.001^*^
Msc	9 (27.3%)	26 (5.1%)	6 (2.9%)		
PhD	4 (12.1%)	168 (33.1%)	64 (31.4%)		
**Graduation year**					
2005–2010	2 (6.1%)	101 (19.9%)	30 (14.7%)	20.330^*^	0.002^*^
2010–2015	11 (33.3%)	86 (17.0%)	35 (17.2%)		
2015–2020	6 (18.2%)	75 (14.8%)	16 (7.8%)		
2020–2024	14 (42.4%)	245 (48.3%)	123 (60.3%)		
**Specialty**					
Endodontics	4 (12.1%)	92 (18.1%)	53 (26.0%)		
Pedodontics	3 (9.1%)	37 (7.3%)	7 (3.4%)	37.444^*^	^MC^p <0.001^*^
Oral- and maxillofacial surgery	0 (0.0%)	12 (2.4%)	0 (0.0%)		
Prosthodontics	0 (0.0%)	21 (4.1%)	15 (7.4%)		
Conservative Dentistry	4 (12.1%)	25 (4.9%)	5 (2.5%)		
Periodontics	17 (51.5%)	166 (32.7%)	60 (29.4%)		
Oral medicine and diagnosis	0 (0.0%)	91 (17.9%)	35 (17.2%)		
Oral Radiology	5 (15.2%)	63 (12.4%)	29 (14.2%)		

χ^2^: Chi square test MC: Monte Carlo

p: p value for Relation between Do you think AI could replace dentists in the future? with demographic data

*: Statistically significant at *p* ≤ 0.05

### Regression analysis

#### Factors affecting usage of AI

Multivariate logistic regression analysis revealed that participants’ use of AI was significantly influenced by their age and speciality. Younger participants, especially those aged (20–25, 25–30), and (30–35) years, were significantly more likely to have attended webinars or lectures about AI in healthcare compared to the reference group (35–40 years old). Conversely, graduates from (2015–2020) and (2020–2024) were less likely to have participated in such educational events (*p* = 0.021 and 0.049). Regarding speciality, endodontists were significantly more likely to report using AI in their practice compared to other specialities (*p* = 0.043). No significant associations were found between AI usage and other specialities, education level, or gender as presented in Table 11.

**Table 11:** Multivariate logistic regression analysis for the parameters affecting usage of AI questions (*n* = 744)

	Using AI for practice (Yes = 152 vs. No = 292)		Do you use any of the current applications of AI-based dental software? (Yes = 159 vs. No = 585)		Have you ever attended any webinar/lecture/course on Artificial Intelligence in healthcare (Yes = 234 vs. No = 510)	
	*p*	OR (LL– UL 95%C.I)	*p*	OR (LL– UL 95%C.I)	*p*	OR (LL– UL 95%C.I)
**Male**	0.595	1.113 (0.750–1.652)	0.627	1.100 (0.748–1.619)	0.874	1.028 (0.732–1.442)
**Age (years)**						
20–25	0.532	3.126 (0.087–111.743)	0.757	1.672 (0.064–43.368)	0.003^*^	36.791 (3.398–398.36)
25–30	0.965	0.924 (0.026–33.188)	0.672	0.496 (0.019–12.824)	0.006^*^	12.943 (2.122–78.950)
30–35	0.908	0.821 (0.029–23.058)	0.709	0.561 (0.027–11.674)	0.014^*^	5.416 (1.408–20.844)
35–40^®^		1.000		1.000		1.000
**Level of education**						
BDs	0.805	1.264 (0.198–8.080)	0.415	2.005 (0.376–10.689)	0.137	2.418 (0.755–7.742)
Msc	0.170	0.172 (0.014–2.133)	0.430	0.453 (0.064–3.231)	0.366	1.650 (0.557–4.889)
PhD^®^		1.000		1.000		1.000
**Graduation year**						
2005–2010^®^		1.000		1.000		1.000
2010–2015	0.420	3.955 (0.140–111.973)	0.436	3.364 (0.159–71.372)	0.252	0.467 (0.127–1.718)
2015–2020	0.189	9.901 (0.324–302.568)	0.207	7.492 (0.327–171.592)	0.021	0.128 (0.023–0.733)
2020–2024	0.319	5.498 (0.192–157.386)	0.385	3.890 (0.182–83.171)	0.049^*^	0.101 (0.010–0.991)
**Specialty**						
Endodontic	0.043^*^	2.246 (1.024–4.925)	0.058	2.091 (0.974–4.485)	0.067	1.809 (0.958–3.415)
Periodontics	0.078	1.966 (0.927–4.168)	0.057	2.022 (0.980–4.172)	0.134	1.561 (0.872–2.792)
Oral medicine and diagnosis	0.679	1.187 (0.527–2.672)	0.695	1.171 (0.532–2.576)	0.896	1.043 (0.550–1.978)
Oral Radiology	0.354	1.489 (0.641–3.458)	0.437	1.385 (0.609–3.153)	0.371	1.361 (0.693–2.671)

OR: Odd`s ratio ^®^: Reference group

C.I: Confidence interval LL: Lower limit UL: Upper Limit

*: Statistically significant at *p* ≤ 0.05

#### Factors affecting perception of a.i. As general views

Gender, age, level of education, and speciality were significant predictors of perceptions of AI as both a new era and a trend in dentistry. Male participants were more likely to perceive AI in dentistry as a current trend (*p* < 0.001) and as a new era (*p* = 0.033). Compared to the reference group, participants in the 25–30 and 30–35 age groups were significantly less likely to view AI as merely a new trend (*p* < 0.001 and 0.002). Interest in studying AI concepts was strongly associated with education level; BDs and MSc holders were significantly more interested than PhD holders (*p* = 0.001 and < 0.001, respectively). The perception of AI as a new era in dentistry was also more prevalent among periodontists (*p* = 0.042). Graduation year did not consistently exhibit significant effects across all items, as presented in Table 12.

**Table 12:** Multivariate logistic regression analysis for the parameters affecting perception of AI questions as general views (*n* = 744)

	Do you think AI is a new era in dentistry? (Yes = 436 vs. No = 308)		Do you think AI is just a new trend? (Yes = 251 vs. No = 493)		Do you feel interested in learning the principles of Artificial Intelligence and its applications in healthcare? (Yes = 374 vs. No = 370)	
	*p*	OR (LL– UL 95%C.I)	*p*	OR (LL– UL 95%C.I)	*p*	OR (LL– UL 95%C.I)
**Male**	0.033^*^	1.459 (1.032–2.064)	< 0.001^*^	1.842 (1.309–2.591)	0.670	0.931 (0.668–1.296)
**Age (years)**						
20–25	0.129	4.703 (0.638–34.642)	0.086	0.148 (0.017–1.314)	0.073	0.130 (0.014–1.214)
25–30	0.736	1.372 (0.218–8.632)	< 0.001^*^	0.024 (0.003–0.160)	0.355	0.393 (0.054–2.846)
30–35	0.230	0.412 (0.097–1.751)	0.002^*^	0.113 (0.028–0.461)	0.140	2.322 (0.759–7.102)
35–40^®^		1.000		1.000		1.000
**Level of education**						
BDs	0.267	1.949 (0.600–6.323)	0.541	0.712 (0.240–2.112)	0.001^*^	19.021 (3.534–102.37)
Msc	< 0.001^*^	18.911 (4.920–72.693)	0.059	0.371 (0.133–1.040)	< 0.001^*^	387.2(45.896–3267.1)
PhD^®^		1.000		1.000		1.000
**Graduation year**						
2005–2010^®^		1.000		1.000		1.000
2010–2015	0.127	3.135 (0.723–13.588)	0.046^*^	4.229 (1.023–17.491)	0.070	0.343 (0.108–1.089)
2015–2020	0.741	0.740 (0.124–4.416)	< 0.001^*^	29.399 (4.580–188.717)	0.122	0.284 (0.058–1.403)
2020–2024	0.997	1.003 (0.160–6.280)	0.920	1.110 (0.147–8.387)	0.285	2.580 (0.453–14.684)
**Specialty**						
Endodontic	0.966	1.013 (0.557–1.842)	0.843	0.941 (0.512–1.727)	0.551	0.835 (0.462–1.509)
Periodontics	0.042^*^	1.717 (1.019–2.893)	0.913	1.029 (0.618–1.712)	0.778	0.927 (0.548–1.569)
Oral medicine and diagnosis	0.279	0.726 (0.407–1.297)	0.211	1.436 (0.814–2.533)	0.225	0.696 (0.388–1.250)
Oral Radiology	0.302	1.396 (0.741–2.630)	0.623	1.168 (0.629–2.168)	0.969	1.012 (0.544–1.884)

OR: Odd`s ratio ^®^: Reference group

C.I: Confidence interval LL: Lower limit UL: Upper Limit

*: Statistically significant at *p* ≤ 0.05

#### Factors affecting perception of AI and its future role in healthcare

Age, speciality, and level of education were all significantly associated with the perception of AI’s potential to enhance dentists’ effectiveness and play a future role in healthcare. Belief in AI’s role in healthcare delivery (*p* < 0.001) and its efficacy benefits (*p* = 0.001 and < 0.001, respectively) was significantly higher among participants with an MSc and a BD degree. Participants aged 25–35 were more likely to believe that AI could improve therapeutic efficacy (*p* = 0.043 and 0.025). Conversely, the perception that AI will significantly impact healthcare was less likely among those who graduated between 2010 and 2020 (*p* = 0.003, 0.005). Furthermore, endodontists and oral medicine specialists were significantly less likely to view AI as beneficial for enhancing practice efficacy (*p* = 0.004 and 0.029, respectively), as presented in Table 13.

**Table 13:** Multivariate logistic regression analysis for the parameters affecting perception of AI questions regarding its future role in healthcare (*n* = 744)

	Do you think that “Artificial Intelligence will play an integral role in delivering healthcare services in the future”? (Yes = 319 vs. No = 425)		Do you think AI could improve the efficacy of dentists in dental practice? (Yes = 294vs. No = 450)	
	*p*	OR (LL– UL 95%C.I)	*p*	OR (LL– UL 95%C.I)
**Male**	0.867	0.971 (0.692–1.364)	0.698	1.067 (0.769–1.482)
**Age (years)**				
20–25	0.998	–	0.137	5.295 (0.58747.761)
25–30	0.837	1.251 (0.147–10.630)	0.043^*^	6.074 (1.05934.832)
30–35	0.012^*^	4.728 (1.416–15.791)	0.025^*^	4.011 (1.18813.538)
35–40^®^		1.000		1.000
**Level of education**				
BDs	< 0.001^*^	40.153 (6.363–253.394)	0.001^*^	7.885 (2.445–25.425)
Msc	0.997	–	< 0.001^*^	23.413 (7.356–74.522)
PhD^®^		1.000		1.000
**Graduation year**				
2005–2010^®^		1.000		1.000
2010–2015	0.003^*^	0.151 (0.042–0.536)	0.074	0.328 (0.097–1.113)
2015–2020	0.005^*^	0.066 (0.010–0.446)	0.002^*^	0.069 (0.012–0.383)
2020–2024	0.998	–	0.136	0.199 (0.024–1.662)
**Specialty**				
Endodontic	0.522	0.815 (0.436–1.523)	0.004^*^	0.407 (0.223–0.745)
Periodontics	0.946	1.020 (0.579–1.795)	0.555	0.853 (0.503–1.447)
Oral medicine and diagnosis	0.997	0.999 (0.539–1.849)	0.029^*^	0.517 (0.286–0.935)
Oral Radiology	0.405	1.320 (0.687–2.534)	0.481	0.799 (0.428–1.491)

OR: Odd`s ratio ^®^: Reference group

C.I: Confidence interval LL: Lower limit UL: Upper Limit

*: Statistically significant at *p* ≤ 0.05

#### Factors affecting perception of AI and impact on speciality and replacement potential

Gender, age, education, and graduation year all influenced participants’ opinions regarding the potential of AI to replace dentists and improve particular specialities. The belief that AI would eventually replace dentists was significantly more common among male participants (*p* = 0.042). Those aged 30–35 were significantly more likely to believe that AI would help advance their fields (*p* < 0.001). Positive perceptions of AI’s role in specialty advancement and replacement potential were significantly associated with lower levels of education; BDs and MSc holders were much more likely to hold these views than PhDs (*p* < 0.001 and 0.001). Moreover, participants who graduated between 2010 and 2024 were significantly less likely to believe that AI would have such effects (*p* < 0.05 for all relevant comparisons). No significant associations were found between speciality and the belief that AI will replace dentists, as presented in Table 14.

**Table 14:** Multivariate logistic regression analysis for the parameters affecting perception of AI questions and impact on speciality and replacement potential (*n* = 744)

	Do you think that AI would facilitate major advancements related to your specialty? (Yes = 253 vs. No = 491)		Do you think AI could replace dentists in the future? (Yes = 33 vs. No = 711)	
	*p*	OR (LL– UL 95%C.I)	*p*	OR (LL– UL 95%C.I)
**Male**	0.144	1.297 (0.915–1.837)	0.042^*^	2.230 (1.028–4.835)
**Age (years)**				
20–25	0.170	6.755 (0.442–103.315)	0.995	–
25–30	0.064	8.444 (0.884–80.666)	0.995	–
30–35	< 0.001^*^	26.963 (6.920–105.059)	0.996	–
35–40^®^				1.000
**Level of education**				
BDs	< 0.001^*^	71.128 (9.381–539.286)	0.047^*^	8.138 (1.029–64.384)
Msc	< 0.001^*^	542.7 (57.365–5134.7)	0.001^*^	24.762 (3.660–167.534)
PhD^®^		1.000		1.000
**Graduation year**				
2005–2010^®^		1.000		1.000
2010–2015	0.001^*^	0.118 (0.034–0.415)	0.023^*^	0.084 (0.010–0.712)
2015–2020	< 0.001^*^	0.009 (0.001–0.075)	0.002^*^	0.015 (0.001–0.207)
2020–2024	0.005^*^	0.026 (0.002–0.328)	0.020^*^	0.009 (0.0002–0.475)
**Specialty**				
Endodontic	0.306	0.714 (0.375–1.360)	0.161	0.359 (0.085–1.504)
Periodontics	0.986	1.005 (0.564–1.791)	0.570	0.732 (0.250–2.146)
Oral medicine and diagnosis	0.339	0.735 (0.391–1.381)	0.996	–
Oral Radiology	0.709	1.134 (0.586–2.191)	0.772	0.825 (0.225–3.030)

OR: Odd`s ratio ^®^: Reference group

C.I: Confidence interval LL: Lower limit UL: Upper Limit

*: Statistically significant at *p* ≤ 0.05

## Discussion

Artificial Intelligence (AI) in dentistry is developing quickly, offering both possibilities and difficulties for clinical treatment and teaching [28]. As digital technologies become more prevalent, understanding how future professionals perceive and adapt to these tools is crucial. Our study aimed to capture these insights from a diverse group of postgraduate dental students in Egypt.

Regarding the demographic data, the majority of participants in the sample were 420 females (56.5%) while males were 324 (43.5%), likewise, Yüzbaşıoğlu [23] observed higher female participation (59% females and 41% males), followed by Murali et al., [17] (73% females and 27% males), and another study [29], reported (71.4% females and 28.9% males). Nonetheless, an identical proportion of participants from both genders (49.2% females and 50.8% males) were reported by Khanagar et al., [18]. One possible explanation for the higher proportion of female respondents in our study is that female students may be more likely to respond to survey-based research on ethical, educational or new technology topics like AI, perhaps as a result of higher conscientiousness or engagement [30].

In the present study, 58.6% of respondents believe that AI will bring in a new era of dental innovation, while 26.5% disagree and 14.9% remain uncertain. Although 42.9% of respondents consider AI essential to healthcare services, only 39.5% agree that AI has the potential to enhance dentists’ effectiveness.

These findings align with the key results reported in a recent systematic review by Dashti et al., 2024 [31], which synthesized data from 13 cross-sectional studies across multiple countries, including India, Saudi Arabia, Turkey, and Pakistan. Their review reported that approximately 72% of dental students believed that artificial intelligence will play a significant role in dentistry in the near future. This perception may be attributed to the fact that AI encompasses a wide range of advanced technologies that are increasingly impacting everyday life. The development of AI enables big data analysis, which supports improved decision-making and yields trustworthy information [28].

About half (50.3%) of the participants in the current study indicated that they would like to learn more about the concepts of artificial intelligence. Likely, a similar study observed a significant increase, with 63.3 and 58.3% of dental students and dentists, respectively [19]. While the percentage of medical and dental students in 63 countries who stated they were interested in the application of AI in daily life was just 15.3% in a prior survey [32].

Despite positive attitudes, significant concerns persist. Many respondents were skeptical about AI’s reliability, with particular apprehension regarding its role in clinical judgment and research originality. These concerns have been noted in multiple international surveys [12, 29, 32] and reflect broader unease about AI’s opacity and the potential for misuse or error.

Dealing with these issues requires a multi-faceted approach; comprehensive AI education in dental curriculum including ethics exposure and ensuring transparency in the application of AI. These actions can bridge the gap between interest and confidence so that future dentists will trust AI systems.

Interestingly, most participants rejected the notion that AI could replace dentists entirely. This skepticism is supported by the recognition that human elements such as empathy, nuanced communication, and ethical reasoning remain irreplaceable in healthcare interactions [33].

The current study found that younger participants and BDS holders are more likely to use AI-based software, as well as showing more optimism about AI’s potential to advance dentistry, whereas PhD holders are more skeptical about its integral role in healthcare. These findings align with previous studies [18, 19], which also report greater interest in AI among younger or less experienced dental professionals. However, differences in educational systems, clinical exposure, and cultural attitudes toward emerging technologies may account for some variation across studies.

These findings may be attributed to the fact that younger participants are exposed to digital technologies and evolving curricula that increasingly incorporate AI-related content. In contrast, the skepticism among PhD holders may reflect their greater clinical experience, familiarity with research standards, and awareness of AI’s current limitations, such as reliability, ethical considerations, and lack of clinical validation.

Furthermore, our results support  the recommendations for structured AI training and clinical verification suggested in the recent literature [1, 2]. The high interest among younger and less experienced dental professionals is consistent with calls to incorporate AI into dental education  and training. Additionally, concerns about clinical reliability and ethical aspects align with the need for strict validation processes before widespread use.

Periodontics (34%) and endodontics (29.6%) stand out as the specialities most aligned with AI developments and the use of AI software, while other fields continue to be marked by uncertainty. These findings align with a previous survey conducted in India, where Endodontics (38%) and Oral Radiology (41%) showed the highest levels of AI adoption, largely due to their reliance on image analysis [17]. Similarly, a study from Saudi Arabia reported that Periodontics (31%) was the leading speciality utilizing AI, driven by digital workflows in implant planning [18]. In contrast, specialities such as Oral Surgery demonstrated lower levels of AI adoption, likely due to the tactile complexity of procedures and limited compatibility with AI-based automation [6]. These results suggest that AI integration is more prevalent in specialities that emphasize diagnostic precision, digital workflows, and data-driven care.

A higher rate of AI usage in Endodontics and Periodontics may indicate the emphasis these disciplines place on diagnostic imaging and quantitative data, areas where AI’s pattern-recognition capabilities provide a significant advantage [34, 35]. The integration of AI with digital dentistry platforms, such as guided implant surgery and dynamic navigation systems, further supports its growing adoption [1, 36, 37]. Moreover, the research-intensive nature of these specialities and AI’s potential to improve the efficiency of time-critical tasks may also contribute to its increased use in these fields [34, 38, 39].

In the present study, the majority (68.1%) doubt that AI might replace dentists, while only 4.4% think it could. It was in accordance with a previous study [20]. Likewise, according to a previous survey [17], 37.78% of dental interns did not agree that this new technology would replace them. While, on the other hand, another study [23], reported that 28.6% of the participants believed that dentists would be replaced by AI.

These results suggest a general consensus that AI will be considered as a supporting role, rather than a replacement for Dentists. The skepticism regarding AI’s ability to completely replace dentists might come from realizing that dental work actually incorporates another level of skill, including clinical decision-making, patient management, and manual dexterity, which are difficult to imitate in AI platforms available today. Additionally, the limited integration of AI into dental education may enhance clinical uncertainty regarding the clinician’s role toward AI.

The accuracy of information generated by AI is a major concern for 83.2% of participants. Two other significant concerns are an over-reliance on technology (78.1%) and a potential loss of originality in research (59.3%). The absence of clinical evidence for AI applications in dentistry was brought up by 77.4% of respondents.

According to Khanagar’s study [18], AI is revolutionary in terms of delivering reliable facts for analytical scientific decision-making. In addition to saving time and lowering the possibility of human error, AI models can be helpful tools for identifying survivors of large-scale disasters and as an extra aid in medico-legal scenarios.

More than half of the responders to the present survey (49.3%) support incorporating AI into graduate or undergraduate education to address knowledge gaps. The majority of participants (73.1%) believe that more clinical studies are necessary to demonstrate the feasibility of AI applications, this was in line with previous studies.

Similarly, according to a prior survey [19], as many as 49.0% of respondents thought that AI education should be taught in dental schools, while the proportion of participants was somewhat higher than that in previous studies [23, 40] as 79.80% and 85.6%, respectively.

Participants advocated for greater integration of AI education into dental curricula and emphasized the need for robust clinical trials to validate AI tools. These findings are aligned with previous studies to include AI literacy in dental education to better prepare students for future practice environments [20, 24, 41].

Ethical considerations play a central place in the responsible integration of AI in dentistry. Globally, there is growing attention to key concerns such as algorithmic bias, data privacy, patient consent, and the transparency of AI decision-making issues that are widely debated in digital health policies. For instance, global digital health strategies emphasize the need for AI systems to be explainable, fair and human-centered, particularly in healthcare settings where flaws systems can directly impact patient outcomes [42]. In the context of dentistry, clear patient communication is essential both when AI tools guide clinical decisions and when such tools have been independently tested for fairness and accuracy. Ethical implementation is not only a technical issue, but also requires regulatory alignment, continuous professional education, and organizational policies with a focus on fairness and accountability.

The multivariate logistic regression analyses provided clear evidence that demographic and professional factors independently impacted dental professionals’ usage and attitude toward AI. In different models, age, speciality, gender, graduation year, and educational level were all significant predictors. Younger participants and those with lower academic qualifications (BD and MSc) showed higher interest in learning about AI and greater optimism regarding its potential to improve dental practice. On the other hand, recent graduates (2010–2024) and PhD holders were considerably more skeptical, especially about AI’s potential to significantly alter healthcare service or replace dentists. While specialities like periodontics and endodontics showed stronger adoption of AI, probably as a result of their reliance on imaging and computerized workflows, male participants were more likely to view AI as a transformative trend.

These findings highlight the critical gap between awareness of AI’s potential and its practical adoption in dentistry. While many practitioners recognize the value of AI, its integration into clinical practice remains limited due to educational, ethical, and practical barriers. Addressing this gap will require structured clinical validation, targeted training, and tailored AI education to build confidence and competence across different practitioner groups. Institutional support and ongoing professional dialogue will also be essential to facilitate the responsible and effective incorporation of AI into dental practice.

This study offers several notable strengths. First, the large and diverse sample of postgraduate dental students from multiple institutions enhances the generalizability of the findings. By addressing a timely and emerging topic AI in dental practice and education the study provides relevant insights into the readiness of future professionals to adopt AI technologies. The structured questionnaire, grounded in existing literature and validated tools, allowed for a comprehensive exploration of participants’ perceptions, usage patterns, and ethical concerns. Additionally, the use of appropriate statistical methods ensured a robust analysis of demographic influences. The ethical conduct of the study, including informed consent and data confidentiality, further strengthens its reliability. Collectively, these elements contribute to a well-rounded understanding of AI’s perceived role and challenges in the context of postgraduate dental education.

### Limitations

This study utilized a cross-sectional survey  design that measures self-reported perceptions and attitudes, which restricts causal inferences and may introduce response bias. The findings may  not be generalizable to other populations or clinical settings due to contextual differences and potential sampling bias. Furthermore, the study focuses on trends in AI awareness and acceptance rather than evaluating the clinical effectiveness or outcomes of AI applications.

While English is the standard language of instruction in all participating dental programs, administrating the survey exclusively in English may be considered a limitation for non-native speakers with varying levels of proficiency, which could have affected their understanding or interpretation of certain survey items.

Additionally, a 3-point forced-choice Likert scale may restrict  response nuance compared to 5 or 7-point scales, it was selected to minimize cognitive burden and ensure consistency across participants with varying familiarity with AI and different levels of language proficiency. Finally, some participants may have experienced survey fatigue  by the number of questions, which could have compromised response quality and validity.

### Implications for practice and education

To responsibly integrate AI into dentistry, a pragmatic and evidence-based approach must guide both clinical implementation and education. This can be achieved through interdisciplinary courses for both undergraduate and postgraduate levels that cover fundamental AI principles, ethical concerns such as bias and privacy, as well as practical experience with AI-based diagnostic systems.

Practical experience with AI-based diagnostic systems should be included in training to prepare future dentists for real-world applications. In clinical practice, AI should be implemented gradually. All AI-assisted decisions must be verified by a dentist via “human-in-the-loop” (HITL) systems to minimize errors and manage legal accountability.

Policymakers should aim to align international regulations, such as those in the U.S. FDA and AI Act within the European Union (EU), and mandate continuous training for skills related to AI for dental professionals. Research should focus on long-term clinical outcomes, cost-effectiveness in low-resource settings, and regular use of reporting guidance such as CONSORT-AI. Funding should also support efforts to reduce disparities in AI access and performance.

### Future directions

To bridge the identified gaps in AI knowledge and readiness, dental education systems must incorporate comprehensive training on artificial intelligence [43]. Prospective clinical trials and real-world studies are essential  to validate the long-term clinical utility and reliability of AI technologies in dentistry. These efforts should involve collaboration with qualified specialists to ensure that AI is integrated in a manner that upholds the  highest standards of privacy, patient safety, and ethical practice. By aligning education with responsible implementation, the dental profession can leverage AI to enhance rather than replace clinical judgment, thereby ensuring patient safety and promoting equitable care.

## Conclusions

In general, younger participants and BDS holders demonstrated greater interest, optimism, and adoption of AI. In contrast, older participants and PhD holders are more suspicious, raising questions regarding replacement, efficacy, and dependability. Periodontics and endodontics emerged as the specialities most aligned with AI advancements, while other fields exhibited greater uncertainty. The study highlights a degree of reluctance to adopt AI, primarily due to concerns about its clinical validity and dependability. Nonetheless, experts with less expertise and younger generations show a greater interest in the exploration of AI applications. To bridge the perception-practice gap, future efforts should prioritize awareness campaigns, focused instructional sessions, and strong clinical validations.

## Electronic supplementary material

Below is the link to the electronic supplementary material.

Supplementary Material 1

## Data Availability

No datasets were generated or analysed during the current study.

## Abbreviations

AI: Artificial Intelligence
BDS: Bachelor of Dental Surgery
PhD: Doctor of Philosophy
LLM: Large Language Model
SPSS: Statistical Package for the Social Sciences
