# Supplementary material for: Perception, usage, and concerns of artificial intelligence applications among postgraduate dental students: cross-sectional study
Source: BMC Med Educ. 2025 Jun 23;25:856. doi: 10.1186/s12909-025-07544-6 (PMC12186392; doi:10.1186/s12909-025-07544-6)
Supplement: Supplementary file 1 — Supplementary Material 1 [file 12909_2025_7544_MOESM1_ESM.docx]

**Demographic data:**

- Gender.
- Age.
- Level of study: BDs - MSc – PhD.
- Specialty.
- Faculty.

**Usage of artificial intelligence (AI):**

1. **Have you ever attended any webinar/lecture/course on Artificial Intelligence in healthcare?**
2. **Do you use or are you familiar with the usage of any of the current applications of AI-based dental software (ORCA Dental AI, Denti AI, VideaHealth, Pearl, Glidewell.io, Smilecloud, DentalXrai Pro, Dental Analytics, Dental monitoring, AssistDent, etc.)?**
3. **Which resource have you used the most to learn about Artificial Intelligence and its applications?**

Websites/ Friends and colleagues/ Journal articles and books/ Formal training (e.g., courses) in Artificial Intelligence/ Medical school lectures / Webinars / Nothing/Others – Please Mention

**Perception of artificial intelligence (AI):**

- - - 1. **Do you think AI is a new era in dentistry?**
      2. **Do you think AI is just a new trend?**
      3. **Do you feel interested in learning the principles of Artificial Intelligence and its applications in healthcare?**
      4. **Do you think that “Artificial** **Intelligence will play an integral role in delivering healthcare services in the future”?**
      5. **Which of the following professions will profit most of the introduction of AI?**

Endodontics / Pedodontics/ Oral- and maxillofacial surgery/ Prosthodontics / Orthodontics/ Conservative Dentistry / Periodontics Other: …………

- - - 1. **Do you think AI could improve the efficacy of dentists in a dental practice?**
      2. **Do you think that your profession is adequately equipped for the application of AI?**
      3. **Do you think AI could replace dentists in a dental practice?**

**Concerns about artificial intelligence (AI) usage:**

**Are you concerned about the reliability of the information provided by AI?**

**Are you afraid of relying too much on AI and not developing critical thinking skills?**

**Are you concerned that using AI would get you accused of plagiarism?**

1. **Are you afraid that using AI would result in a lack of originality in your university assignments and duties?**
2. **Are you afraid that the use of AI would be a violation of academic and university policies?**
3. **Are you afraid of becoming too dependent on technology like AI?**
4. **Are you enthusiastic about using technology such as AI for learning and research?**
5. **Do you think that AI is an important tool for academic success?**
